# Follistatin Alleviates Synovitis and Articular Cartilage Degeneration Induced by Carrageenan

**DOI:** 10.1155/2014/959271

**Published:** 2014-12-09

**Authors:** Jun Yamada, Kunikazu Tsuji, Kazumasa Miyatake, Yu Matsukura, Kahaer Abula, Makiko Inoue, Ichiro Sekiya, Takeshi Muneta

**Affiliations:** ^1^Department of Joint Surgery and Sports Medicine, Tokyo Medical and Dental University, Tokyo 113-8519, Japan; ^2^Department of Cartilage Regeneration, Tokyo Medical and Dental University, 1-5-45 Yushima, Bunkyo-ku, Tokyo 113-8519, Japan; ^3^International Research Center for Molecular Science in Tooth and Bone Diseases (Global Center of Excellence Program), Tokyo Medical and Dental University, Tokyo 113-8519, Japan; ^4^Department of Plastic and Reconstructive Surgery, Tokyo Medical and Dental University, Tokyo 113-8519, Japan; ^5^Center for Stem Cell and Regenerative Medicine, Tokyo Medical and Dental University, Tokyo 113-8519, Japan

## Abstract

Activins are proinflammatory cytokines which belong to the TGF*β* superfamily. Follistatin is an extracellular decoy receptor for activins. Since both activins and follistatin are expressed in articular cartilage, we hypothesized that activin-follistatin signaling participates in the process of joint inflammation and cartilage degeneration. To test this hypothesis, we examined the effects of follistatin in a carrageenan-induced mouse arthritis model. Synovitis induced by intra-articular injection of carrageenan was significantly alleviated by preinjection with follistatin. Macrophage infiltration into the synovial membrane was significantly reduced in the presence of follistatin. In addition, follistatin inhibited proteoglycan erosion induced by carrageenan in articular cartilage. These data indicate that activin-follistatin signaling is involved in joint inflammation and cartilage homeostasis. Our data suggest that follistatin can be a new therapeutic target for inflammation-induced articular cartilage degeneration.

## 1. Introduction

Osteoarthritis (OA) is considered to be a multifactorial disease with risk factors such as inflammation, aging, menopause, obesity, genetic background, and joint instability [1]. Clinical manifestations of OA may include joint pain, tenderness, stiffness, creaking, locking of joints, and local inflammation. Currently, no curative treatment for OA has been developed, and the major therapeutic strategies for OA are based on conservative treatments and surgical intervention (joint replacement). For the conservative treatments, most guidelines include recommendations for muscle exercise with medications to increase joint stability and to relieve joint inflammation and pain [2, 3]. For example, the 2014 OARSI guidelines for nonsurgical management of knee osteoarthritis indicated that land-based exercise (such as t'ai chi), water-based exercise, and strength training are all recommended to improve pain and physical function in knee OA [4]. For the pharmacological interventions, the guideline indicated that both oral nonselective and COX2-selective NSAIDS (nonsteroidal anti-inflammatory drugs) as well as topical NSAIDS are conditionally recommended to improve the symptoms in knee OA [4]. In addition to their effects on joint inflammation and pain relief, recent animal research has shown that some NSAIDs have anticatabolic and antiapoptotic effects on articular cartilage [5, 6]. These findings suggest that control of joint inflammation may be an important therapeutic target to avoid OA progression and improve OA symptoms.

In this study, we examined the anti-inflammatory and anticatabolic effects of follistatin on a carrageenan-induced mouse arthritis model. Follistatin is an extracellular decoy receptor for the proinflammatory cytokine, activin (inhibin*β*) [7]. It is a glycosylated polypeptide structurally unrelated to activins and was originally isolated from ovarian fluid, where it mimics the action of inhibin on follicle stimulating hormone (FSH) secretion. Since binding affinity of follistatin to activin is high, follistatin is considered to neutralize the actions of activins almost irreversibly [7].

Activin was originally isolated from gonadal fluids based on the ability to induce FSH secretion from the pituitary gland. Activins are homo- or heterodimeric cysteine knot proteins belonging to the TGF*β* superfamily. To date, 4 genes have been identified in the activin family (inhibin*β*A, *β*B, *β*C, and *β*E) and 3 dimeric proteins (activin A (*β*A/*β*A), activin B (*β*B/*β*B), and activin AB (*β*A/*β*B)) have been studied in detail [8]. Subsequent analyses indicated that, in addition to being integral to reproductive physiology, activins are involved in many physiological processes such as embryonic development, tissue homeostasis, and tissue repair [9, 10]. Furthermore, recent reports indicated that the release of activins is involved in the acute inflammatory processes and immune responses in various clinical contexts such as sepsis, liver fibrosis, acute lung injury, asthma, wound healing, ischemia-reperfusion injury, and rheumatoid arthritis [11, 12]. In acute systemic inflammatory situations, activins surge in the very early time point after the challenge. Jones et al. reported that administration of 50 *μ*g of lipopolysaccharide (LPS) into the jugular vein of Corriedale ewes increased plasma activin A levels within one hour after LPS injection [13, 14]. The surge of activins after LPS injection occurred at almost the same time or just prior to the surge of key proinflammatory cytokines such as TNF*α* and IL6. Importantly, recent reports confirmed the inhibitory effects of follistatin on activin action during inflammatory processes in various mouse models. Dohi et al. reported that administration of follistatin neutralized the action of activin and alleviated symptoms in a mouse colitis model [15]. Hardy et al. suggested that the balance between activin A and follistatin is a determinant of severity of allergic inflammation or tissue phenotypic shift in asthma [16]. Jones et al. showed that treatment with follistatin altered the expression profiles of proinflammatory cytokines and increased survival after administration of a lethal dose of LPS [17]. Their data strongly suggest the crucial roles of activin in the inflammatory response and that follistatin has significant therapeutic potential to reduce the severity of inflammatory diseases. Since expression of both activins and follistatin is observed in the joint tissues [12, 18], we hypothesized that follistatin may function as an anti-inflammatory cytokine in the joint.

To test this hypothesis, we examined the effect of recombinant mouse follistatin protein in the carrageenan-induced arthritis model. In this study, we showed that administration of follistatin in arthritic mice significantly alleviates synovial inflammation and inhibits proteoglycan loss from articular cartilage. Our data suggest the involvement of activin signaling in the process of joint inflammation and that follistatin may have therapeutic potential to alleviate OA symptoms.

## 2. Materials and Methods

### 2.1. Animals and Materials

Animal care and all experiments were conducted in accordance with the guideline of the Animal Committee of Tokyo Medical and Dental University. C57BL/6J mice were purchased from ORIENTAL YEAST Co., Ltd. (Tokyo, Japan). Mice were housed under a 12-hour light-dark cycle and allowed food and water* ad libitum*. *λ*-Carrageenan was purchased from Sigma-Aldrich (St. Louis, MO). Recombinant mouse follistatin was purchased from R&D Systems Inc. (Minneapolis, MN). Rat anti-mouse monoclonal F4/80 antibody was purchased from AbD Serotec (Oxford, UK).

### 2.2. Carrageenan-Induced Arthritis Model

**Figure 1:**
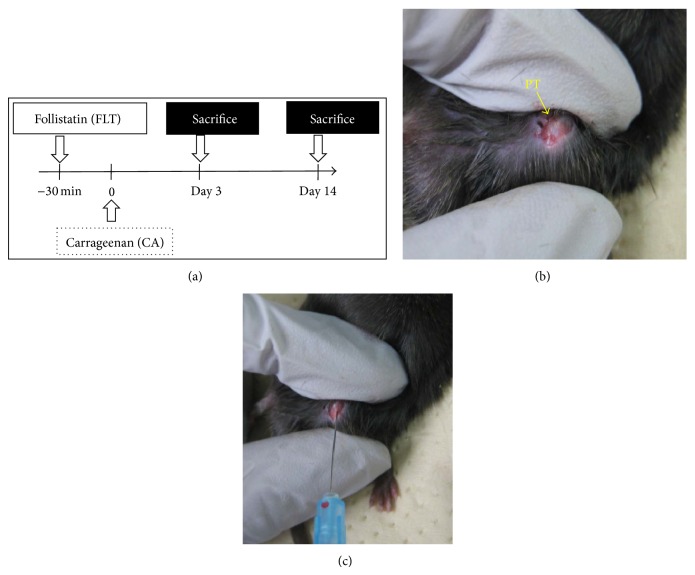
Carrageenan-induced arthritis model. (a) Experimental design of carrageenan-induced arthritis model. Twenty-eight male mice (12-week-old) were registered in this study. Mice were randomly divided into two groups: carrageenan group (CA) and carrageenan + follistatin (CA + FLT) group (*n* = 14/group). Histological analyses were performed at days 3 and 14. (b, c) Procedure of intra-articular injection of carrageenan. PT: patellar tendon.

Thirty-three male mice (12-week-old) were registered in this study. Mice were randomly divided into three groups: carrageenan group (CA), carrageenan + follistatin (CA + FLT) group (*n* = 14/group), and follistatin group (FLT, *n* = 5, Figure 1(a)). Mice were anesthetized by inhalation of 5% isoflurane in oxygen. A small skin incision was created to expose the knee joint (Figure 1(b)). First, 19 mice (14 of CA + FLT and 5 of FLT) were injected with follistatin (25 ng in 5 *μ*L of saline) in the left knee joint (Figure 1(a)). To inject, Hamilton syringes with 31-gauge needles were used. Needles were inserted through the lateral infrapatellar area toward the intercondylar space of the femur in a deep knee-flexed position (Figure 1(c)). After 30 min, all the mice in CA and CA + FLT groups were injected with *λ*-carrageenan solution (30 *μ*g in 5 *μ*L saline) into the left knee joint through the lateral margin of the patellar tendon to induce joint inflammation. Seven mice in CA and CA + FLT groups and 5 in FLT group were sacrificed at day 3, and the remaining mice in each group (*n* = 7) were sacrificed at day 14 after injection (Figure 1(a)). Right knees were kept intact as internal controls.

### 2.3. Histological Analyses of the Knee Joint

Left knee joints were dissected, fixed in 4% PFA (paraformaldehyde; Sigma-Aldrich, MO) in the fixed angle (30 degrees), decalcified in 20% EDTA (ethylenediaminetetraacetic acid in PBS, pH 7.4), and embedded in paraffin. Five *μ*m thick sagittal sections of medial weight-bearing regions were prepared, and sections of equivalent position from each mouse were selected by the shape of the growth plate and further analyzed in this study. Each section was stained with safranin-O/fast green or hematoxylin and eosin (H&E) for the histological evaluation of cartilage degeneration and joint inflammation. We analyzed two other sections 100 *μ*m away (on both the medial and lateral sides) from the analyzed sections of each mouse and observed similar results (data not shown).

To evaluate the early stage of articular cartilage degeneration, which is indicated by the loss of proteoglycan from articular cartilage, we employed a semiquantitative scoring system described by Coles et al. with minor modification as described in (*Modified Coles Score (Coles et al., Arthritis & Rheumatology, 2010)*) [19]. Histological scores of articular cartilage from femurs and tibiae were evaluated and total scores were compared between the two groups at each time point.

*Modified Coles Score (Coles et al., Arthritis & Rheumatology, 2010) [19]*

1. Articular cartilage structure is as follows:
   0 indicates normal;
   1 indicates some surface irregularities, no fibrillation;
   2 indicates severe surface irregularities and undulation;
   3 indicates fibrillation, clefts, or cartilage loss into superficial zone;
   4 indicates fibrillation, clefts, or cartilage loss into middle zone.
2. Surface layer morphology is as follows:
   0 indicates smooth;
   1 indicates some small irregularities;
   2 indicates moderately roughened or enlarged;
   3 indicates severely enlarged, cellular infiltrate.
3. Pericellular loss of safranin-O staining is as follows:
   0 indicates no pericellular loss of staining;
   1 indicates proteoglycan loss observed in less than 30% of articular surface;
   2 indicates proteoglycan loss observed in between 30% and 60% of articular surface;
   3 indicates proteoglycan loss observed in more than 60% of articular surface.

**Figure 2:**
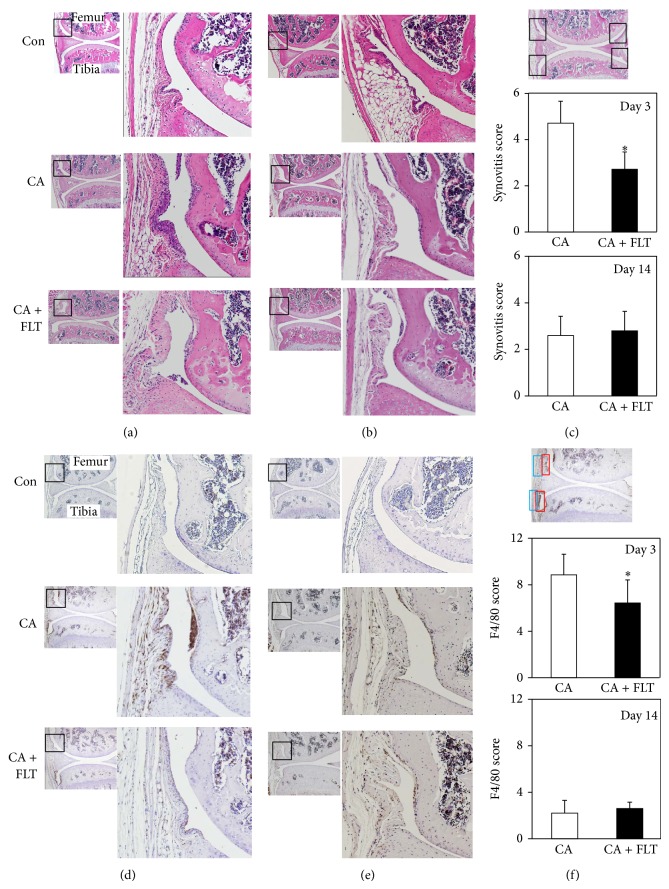
Histological analyses of synovial inflammation induced by carrageenan. (a, b) H&E staining of sagittal sections of mice knee joints at day 3 (a) and day 14 (b) after carrageenan injection. Boxed areas in the left panels are magnified and presented in the right panels. CA: carrageenan group; CA + FLT: carrageenan + follistatin group; Con: control group. Magnification: left ×40, right ×100. (c) Semiquantitative analyses of synovial inflammation. The severity of synovial inflammation was evaluated according to the synovitis scoring system (*Synovitis Score *(*Blom et al., osteoarthritis and cartilage, 2004* [20])). The synovitis scores are indicated as the total of four different areas in the knee joint as denoted in upper panel. (^*^ *P* < 0.05). (d, e) Immunohistochemical analyses of macrophage infiltration into synovial membrane at day 3 (d) and at day 14 (e) after carrageenan injection. Sagittal sections of mice knee joints were stained with F4/80 antibody. Sections were counterstained with hematoxylin. Boxed areas in the left panels are magnified and presented in the right panels. CA: carrageenan group; CA + FLT: carrageenan + follistatin group; Con: control group. Magnification: left ×40, right ×100. (f) Semiquantitative analyses of the degree of F4/80-positive macrophage infiltration into synovial membrane (*F4/80 Score* (*Willis et al., JOI, 2012* [22])). The scores were indicated as the total of four different areas of the synovial membrane (superficial layer (boxed by red) and deep layer (boxed by blue) of suprapatellar or infrapatellar region) as described in the upper panel (^*^ *P* < 0.05).

The severity of synovial inflammation was evaluated according to the synovitis scoring system described by Blom et al. (summarized in (*Synovitis Score (Blom et al., osteoarthritis and cartilage 2004)*)) [20]. Inflammatory cells were discriminated based on their morphology. The synovitis scores were indicated as the total of four different areas in the knee joint as denoted in Figure 2(c) (upper panel).

*Synovitis Score (Blom et al., osteoarthritis and cartilge, 2004) [20]*

 0 indicates no changes compared to normal joints;
 1 indicates thickening of the synovial lining and some influx of inflammatory cells;
 2 indicates thickening of the synovial lining and intermediate influx of inflammatory cells;
 3 indicates profound thickening of the synovial lining (more than four cell layers) and maximal observed influx of inflammatory cells.

### 2.4. Immunohistochemical Analyses of Macrophage Infiltration into Synovial Membrane

Mouse macrophage specific F4/80 staining was performed as described by Blom et al. [21]. Briefly, sections were deparaffinized, rinsed with PBS, and then incubated in 3% H_2_O_2_ in methanol for 30 min at room temperature to deactivate endogenous peroxidase. After blocking with normal rabbit serum (Vector Laboratories, CA) for 30 min, sections were incubated with rat anti-mouse monoclonal F4/80 antibody (1 : 2000 dilution) at 4°C overnight. After extensive washing with PBS 0.1% tween 20, signals were visualized by the VECTASTAIN ABC kit (Vector Laboratories, CA). The degree of F4/80-positive macrophage infiltration into synovial membrane, which represents the severity of synovitis, was scored as described by Willis et al. (*F4/80 Score (Willis et al., JOI, 2012)*) [22]. The scores were indicated as the total of four different areas of the synovial membrane (superficial layer and deep layer of suprapatellar or infrapatellar region) as described in Figure 2(f).

*F4/80 Score (Willis et al., JOI, 2012) [22]*

 0 indicates no staining;
 1 indicates minimal to few faintly positive cells;
 2 indicates scattered single positive cells;
 3 indicates clusters of two or more positive cells;
 4 indicates larger clusters of positive cells, multifocal to coalescing.

### 2.5. Statistical Analysis

Statistical analyses were performed using Mann-Whitney *U* test and *P* values less than 0.05 were considered significant.

## 3. Results

### 3.1. Follistatin Relieved Synovial Inflammation Induced by Carrageenan

Since it was reported that intra-articular injection of carrageenan initiates a localized synovial inflammatory response that causes articular cartilage degeneration [23], we examined the anti-inflammatory effects of follistatin at day 3 after carrageenan injection. As shown in Figure 2(a), single intra-articular injection of carrageenan induced synovial hyperplasia and increased cellularity in the synovial membrane at day 3. Blom's synovitis score indicated that carrageenan significantly enhanced synovitis (Figure 2(c), middle panel). Interestingly, preinjection of follistatin significantly reduced infiltration of inflammatory cells into the synovial membrane and alleviated synovitis, although fibrotic changes in the synovial membrane were still observed (Figure 2(a)). These acute inflammatory responses induced by carrageenan injection were almost quenched at 14 days after carrageenan challenge (Figures 2(b) and 2(c) lower panels).

To further elucidate the physiological roles of follistatin in alleviation of synovial inflammation, we analyzed macrophage infiltration after carrageenan challenge. As shown in Figures 2(d) and 2(e), significant numbers of F4/80-positive macrophages migrated into both superficial and deep regions of synovial membrane at day 3 after carrageenan challenge. In contrast, preincubation with follistatin greatly reduced macrophage accumulation in both regions of the synovial membrane. These effects were specific to the 3rd day after carrageenan challenge as we did not observe any significant alteration in macrophage infiltration between control, CA, and CA + FLT groups at day 14 (Figures 2(d)–2(f)).

### 3.2. Follistatin Alleviates Articular Cartilage Degeneration Induced by Carrageenan

**Figure 3:**
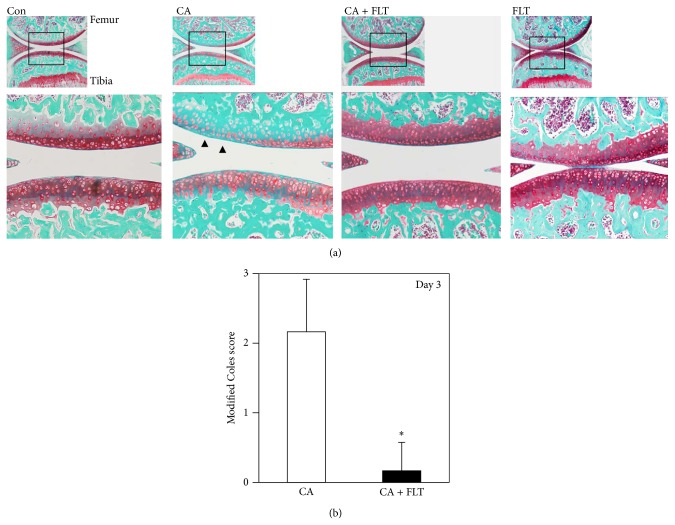
Histological analyses of articular cartilage degeneration induced by carrageenan at day 3. (a) Safranin-O staining of sagittal sections of mice knee joints at day 3 after carrageenan injection. Boxed areas in the top panels are magnified and presented in the bottom panels. CA: carrageenan group; CA + FLT: carrageenan + follistatin group; Con: control group; FLT: follistatin group. Magnification: top ×40, bottom ×100. Arrowhead indicates the region where proteoglycan erosion is observed. (b) Semiquantitative analyses of articular cartilage degeneration. The degree of articular cartilage degeneration was semiquantitatively evaluated (*Modified Coles Score *(*Coles et al., Arthritis & Rheumatology, 2010*)) [19]. Histological scores of articular cartilage from femurs and tibiae were evaluated, respectively, and total scores were compared between the two groups (^*^ *P* < 0.05).

As reported previously [23], single intra-articular injection of carrageenan reduced proteoglycan content in both femur and tibia articular cartilage at day 3, indicated by the reduced dyeability of safranin-O (Figure 3(a)) in comparison to control and CA groups. Modified Coles score indicated that intra-articular injection of carrageenan significantly induced articular cartilage degeneration at this time point (Figure 3(b)). However, we did not observe any significant alterations in the articular surface structures such as fissure formation, increased cellularity, and hypertrophic differentiation of articular chondrocytes at this stage (Figure 3(a)). Interestingly, preinjection of follistatin preserved safranin-O dyeability and improved the modified Coles score almost to the same level as that of control (Figures 3(a) and 3(b)). Follistatin itself does not seem to have effects on proteoglycan metabolism since no apparent histological alteration was observed between the control and FLT groups (Figure 3(a)).

**Figure 4:**
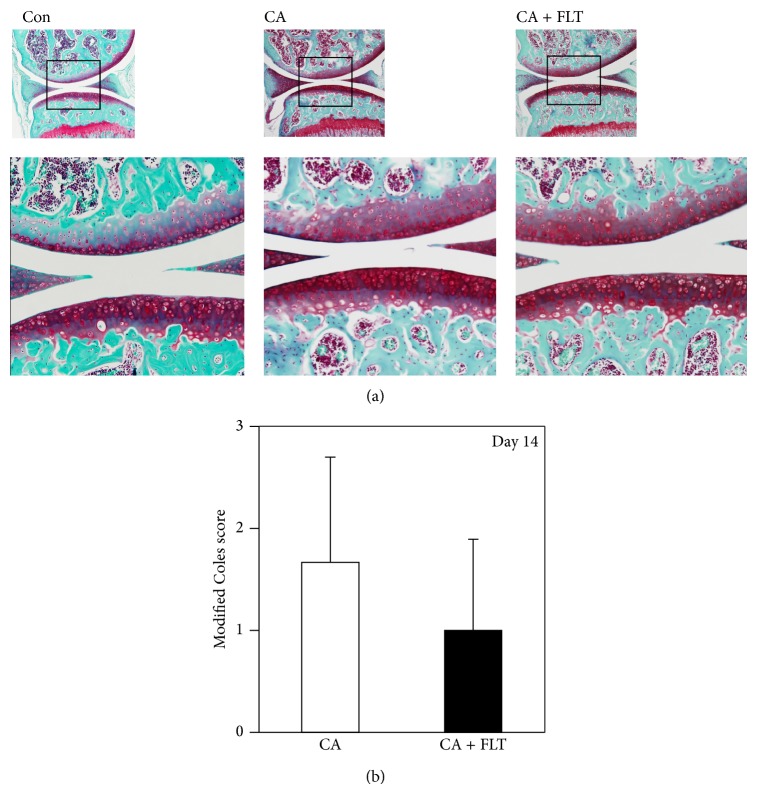
Histological analyses of articular cartilage degeneration induced by carrageenan at day 14. (a) Safranin-O staining of sagittal sections of mice knee joints at day 14 after carrageenan injection. Boxed areas in the top panels are magnified and presented in the bottom panels. CA: carrageenan group; CA + FLT: carrageenan + follistatin group; Con: control group. Magnification: top ×40, bottom ×100. (b) Semiquantitative analyses of articular cartilage degeneration. The degree of articular cartilage degeneration was semiquantitatively evaluated (*Modified Coles Score *(*Coles et al., Arthritis & Rheumatology, 2010*) [19]). Histological scores of articular cartilage from femurs and tibiae were evaluated, respectively, and total scores were compared between the two groups (^*^ *P* < 0.05).

At day 14, after carrageenan challenge, safranin-O dyeability was reversed to the control levels in the CA group and we did not observe any significant alteration in the articular surface structure between control, CA, and CA + FLT groups (Figures 4(a) and 4(b)).

## 4. Discussion

In this study, we showed that follistatin significantly alleviated carrageenan-induced synovial inflammation and articular cartilage degeneration. Detailed histological analyses indicated that preinjection of follistatin significantly reduced macrophage infiltration into the synovial membrane and protected articular cartilage from proteoglycan erosion at day 3 after carrageenan injection. Our data suggest the potential for follistatin as a novel anti-inflammatory and anticatabolic drug for OA treatment.

Carrageenan is a sulphated mucopolysaccharide extracted from the seaweeds* Chondrus *spp. and* Gigartina *spp., commonly known as Irish moss or carrageen moss from red Scottish seaweed [24]. It is known for its remarkable capacity to stimulate local inflammation dominated by intense macrophage aggregation and by fibroblastic proliferation [25, 26]. Molecular action of carrageenan is mediated by members of the family of innate immune receptors, such as TLR2 and TLR4 (Toll-like receptors 2 and 4). TLR2 and TLR4 are expressed in a broad range of mammalian cells including epithelial/endothelial cells and immune-related cells such as monocytes, macrophages, and T-cells [27, 28]. Upon recognition of their cognate ligands, TLRs form homo- or heterodimers and activate intracellular signals through recruitment of different combinations of TIR-domain-containing adaptor proteins. The major components of these adaptor molecules include MyD88 (myeloid differentiation factor 88) and MAL/TIRAP (MyD88-adaptor-like) [29]. The interaction between MyD88 and IRAKs (IL1 receptor associated kinase) induces the formation of macromolecular complexes that ultimately impinge on TAK1 (TGF*β* activated kinase 1) and lead to activation of NF*κ*B, which has pivotal roles in the induction of various cytokines and chemokines such as IL1*β*, IL6, and IL8 in various cell types [30].

Carrageenan-induced arthritis is a well-established experimental animal model to investigate inflammation-mediated articular cartilage degradation in rodents and rabbits [31]. It is reported that single intra-articular injection of carrageenan initiates a localized synovial inflammatory response, which is indicated by synovial hyperplasia and macrophage accumulation, and decreases both the proteoglycan content and the rate of* de novo* proteoglycan synthesis in the articular cartilage [23]. Although it is not fully elucidated, the TLR-interleukin axis is likely to be involved in the process of joint inflammation and cartilage degeneration induced by carrageenan, since synovial cells also express both TLR2 and TLR4 and their expression levels are increased in RA (rheumatoid arthritis) synovium [32].

Activin levels are reported to surge in the very early stage during the inflammatory response, which is almost the same time or slightly prior to the induction of proinflammatory cytokines TNF*α* and IL6 [13, 14]. Although there is no evidence that proves the direct involvement of activins on the upregulation of proinflammatory cytokines and chemokines induced by TLR signals, we consider that it is likely because previous studies reported that activin A release after LPS exposure was significantly suppressed in mice lacking MyD88 and that follistatin significantly inhibited the surge of serums TNF*α*, IL1*β*, and IL6 after LPS challenge in mice [17]. We speculate that our data correlates with these findings since our synovitis score was significantly improved in the CA + FLT group at day 3 when compared to that in the CA group (Figure 2(c)).

Interestingly, synovial fibrosis was still observed in the CA + FLT group (Figure 2(a)) while F4/80-positive macrophage infiltration into the synovial membrane was significantly inhibited at day 3 (Figure 2(d)). Synovial fibrosis is a major contributor to joint stiffness in OA, and TGF*β* is considered to play central roles in the onset and persistence of this process. Seki et al. reported that the TLR4-MyD88-NF*κ*B dependent pathway enhances TGF*β* signaling through downregulation of Bambi (BMP and activin membrane-bound inhibitor), a pseudoreceptor for TGF*β*/BMP family molecules [33]. It is still unclear if activin signal negatively contributes to the process of synovial fibrosis. Our data suggest that follistatin may specifically interfere with the TLR4-MyD88-NF*κ*B dependent pathway of proinflammatory cytokine expressions but may not be involved in the TLR-Bambi-TGF*β* pathway.

In this study, we also showed that follistatin significantly inhibited proteoglycan erosion induced by carrageenan. Accumulating data indicate that cartilage matrix degrading enzymes such as ADAMTSs and MMPs are regulated by proinflammatory cytokines such as IL1*β*. Tian et al. reported that ADAMTS4 expression in nucleus pulposus (NP) cells was enhanced by IL1*β* through MAPK and NF*κ*B activation [34]. Kataoka et al. reported that ADAMTS4 expression in synoviocytes was enhanced by IL1*β* and this upregulation was reversed by synthetic inhibitors for p38MAPK (SB239063) and JNK (SP600125) [35]. Matsushita et al. reported that IL1*β* induced MMP1, MMP2, MMP9, MMP13, and ADAMTS5 in human chondrocytes [36]. Our data suggest that chondroprotective effects of follistatin may also be through the downregulation of TLR-MyD88-NF*κ*B-IL1*β* signaling pathways.

Molecular mechanisms by which activins modulate TLR-MyD88-NF*κ*B signal pathways are still unclear. Several recent publications reported negative cross talk between TGF*β*/BMP and TLR-MyD88-NF*κ*B signaling pathways. Lee et al. reported that K48-linked polyubiquitination and degradation of MyD88 were induced by TGF*β*, which was mediated by Smad6-specific recruitment of Smurf E3 ligases [37]. Huang et al. reported that TLR4 dependent NF*κ*B activation inhibited BMP2-induced phosphorylation of Smad1/5/8 [38]. Based on our study, we speculate that signals mediated by activins may positively regulate TLR-MyD88-NF*κ*B dependent proinflammatory cytokine expression in the joint. Further studies are required to elucidate the molecular mechanisms by which activins/follistatin signals regulate joint inflammation and articular cartilage metabolism.

In summary, we demonstrated that follistatin significantly alleviated synovial inflammation and articular cartilage degeneration induced by carrageenan. We expect that follistatin or other extracellular activin blocking reagents such as ActRIIa-Fc and ActRIIb-Fc can be utilized as potent inhibitors for inflammation-mediated articular cartilage degeneration.
